# Circulating MicroRNAs: Association with Lung Function in Asthma

**DOI:** 10.1371/journal.pone.0157998

**Published:** 2016-06-30

**Authors:** Alvin T. Kho, Sunita Sharma, Joshua S. Davis, Joseph Spina, Dagnie Howard, Kevin McEnroy, Kip Moore, Jody Sylvia, Weiliang Qiu, Scott T. Weiss, Kelan G. Tantisira

**Affiliations:** 1 Children’s Hospital Informatics Program, Boston Children’s Hospital and Harvard Medical School, Boston MA 02115, United States of America; 2 Channing Division of Network Medicine, Brigham and Women’s Hospital and Harvard Medical School, Boston, MA 02115, United States of America; 3 Division of Pulmonary and Critical Care Medicine, Brigham and Women’s Hospital and Harvard Medical School, Boston, MA 02115, United States of America; 4 Pulmonary and Critical Care Unit, Massachusetts General Hospital and Harvard Medical School, Boston, MA 02115, United States of America; 5 Oregon Health & Science University, Portland, OR 97239, United States of America; 6 Partners Personalized Medicine, Partners HealthCare System, Boston, MA 02115, United States of America; 7 Division of Pulmonary Sciences and Critical Care Medicine, University of Colorado School of Medicine, Aurora, CO 80045, United States of America; Cincinnati Children's Hospital Medical center, UNITED STATES

## Abstract

**Background:**

MicroRNAs are key transcriptional and network regulators previously associated with asthma susceptibility. However, their role in relation to asthma severity has not been delineated.

**Objective:**

We hypothesized that circulating microRNAs could serve as biomarkers of changes in lung function in asthma patients.

**Methods:**

We isolated microRNAs from serum samples obtained at randomization for 160 participants of the Childhood Asthma Management Program. Using a TaqMan microRNA array containing 754 microRNA primers, we tested for the presence of known asthma microRNAs, and assessed the association of the individual microRNAs with lung function as measured by FEV_1_/FVC, FEV_1_% and FVC%. We further tested the subset of FEV_1_/FVC microRNAs for sex-specific and lung developmental associations.

**Results:**

Of the 108 well-detected circulating microRNAs, 74 (68.5%) had previously been linked to asthma susceptibility. We found 22 (20.3%), 4 (3.7%) and 8 (7.4%) microRNAs to be associated with FEV_1_/FVC, FEV_1_% and FVC%, respectively. 8 (of 22) FEV_1_/FVC, 3 (of 4) FEV_1_% and 1 (of 8) FVC% microRNAs had functionally validated target genes that have been linked via genome wide association studies to asthma and FEV1 change. Among the 22 FEV_1_/FVC microRNAs, 9 (40.9%) remain associated with FEV_1_/FVC in boys alone in a sex-stratified analysis (compared with 3 FEV_1_/FVC microRNAs in girls alone), 7 (31.8%) were associated with fetal lung development, and 3 (13.6%) in both. Ontology analyses revealed enrichment for pathways integral to asthma, including PPAR signaling, G-protein coupled signaling, actin and myosin binding, and respiratory system development.

**Conclusions:**

Circulating microRNAs reflect asthma biology and are associated with lung function differences in asthmatics. They may represent biomarkers of asthma severity.

## Introduction

Asthma affects ~23 million individuals in the United States and ~300 million individuals worldwide[1] with rising prevalence. Over US$50 billion were spent on asthma in the U.S.A. in 2011. Despite this, progress toward novel diagnostic markers and therapies for asthma has been slow. MicroRNAs are single-stranded RNA molecules of 19–24 nucleotides in length. MicroRNAs are post-transcriptional regulators that bind to complementary sequences (often imperfectly) on target messenger RNA transcripts (mRNAs), usually resulting in translational repression or gene silencing[2]. In addition to regulating gene expression, microRNAs control a wide range of cell processes, including cell differentiation and growth, development, metabolism, signaling and apoptosis, as well as disease processes linked with cancer and inflammation[3]. Although human studies exploring microRNA differences between asthmatic patients and healthy controls have been performed, comprehensive assessment of microRNAs in inflammatory diseases, including asthma, remains largely unexplored.

With proper storage, circulating microRNAs are stable over many years[4–8]. The stability of circulating microRNA is characterized by its resistance to RNase activity, extreme pH and temperature[9–11], largely because they are bound to proteins, lipoproteins or are within exosomes[4, 12–16]. The combination of stability and measurability supports circulating microRNAs as noninvasive, sensitive biomarkers of disease[9, 10, 17]. In inflammatory diseases, circulating microRNAs likely arise from 2 sources: activated immune cells and tissues damaged by the immune attack[17]. To date, 2 asthma studies of limited sample sizes have analyzed circulating microRNAs[18, 19] and identified 30 microRNAs of which 4 been linked to asthma in previous studies of airway cells.

MicroRNA studies in asthma have mainly focused on asthma diagnosis. Quantitative severity measures may be more specific to asthma pathobiology, as they are not subject to potential diagnostic bias or misclassification. In this study, we investigated the association of circulating microRNAs in 160 asthmatic children with lung function measures as quantifiers of asthma severity at the time of randomization of the Childhood Asthma Management Program (CAMP) clinical trial. We focused on measures of lung function due to their easy measurement, reproducibility and role as part of current asthma treatment guidelines[20]. Given that this is the first large scale analysis of circulating microRNAs in asthma severity, we note that a significant proportion of microRNAs detected in the serum of childhood asthmatics have previously been associated with asthma in tissue studies, providing direct evidence of biological significance. Below, we describe the association of microRNAs with the degree of lung function impairment and the biochemical pathways predicted to be affected by these microRNAs, which together may yield novel insights into asthma pathogenesis.

## Materials and Methods

### Study population and samples

CAMP was a multicenter, randomized, double-blinded clinical trial testing the safety and efficacy of inhaled budesonide, nedocromil and placebo in 1041 children with mild to moderately severe asthma over a 4.3-year average. The trial design and methodology have been published[21, 22]. Entry criteria included asthma symptoms and/or medication use for ≥6 months in the previous year and airway responsiveness with PC_20_ ≤ 12.5 mg/ml. Spirometry was performed on a Collins Stead-Wells dry-seal Survey III spirometer[21]. At least 3 acceptable maneuvers meeting American Thoracic Society (ATS) standards were required, with at least 2 reproducible (forced expiratory volume in one second (FEV_1_) and forced vital capacity (FVC) within 5% of best) maneuvers required for each test. Outcome measures of lung function included FEV_1_ and FVC as a percent of predicted (FEV_1_% and FVC%) and FEV_1_/FVC.

Blood serum from 160 CAMP subjects obtained at randomization were microRNA profiled with 13 subjects profiled twice to model technical replicability. In order to limit the known effects of race on microRNA expression[23, 24], the subjects were limited to self-identified non-Hispanic Caucasians. The subject characteristics are listed in Table 1. The CAMP Genetics Ancillary Study was approved by the Brigham and Women's Hospital Internal Review Board, and informed written consent/assent was obtained from all participants and their guardians.

**Table 1:** CAMP study population characteristics. Values shown as mean ± one standard deviation.

Characteristics	CAMP subjects
**N**	160
**Sex—Male (%)**	87 (54.4%)
**Age, year**	8.83 ± 2.12
**FEV**_**1**_**%**	93.35 ± 14.72
**FVC %**	106.13 ± 13.51
**FEV**_**1**_**/FVC**	78.73 ± 8.71

Human fetal lung tissue from 15 subjects aged 67 to 115 days post conception with non-smoking mothers were acquired through the tissue retrieval program of the National Institute of Child Health and Development at the University of Maryland Brain and Tissue Bank for Developmental Disorders (Baltimore, MD), and the University of Washington Center for Birth Defects Research (Seattle, WA) as previously described[25]. This tissue collection has been designated an institutional review board (IRB)-exempt protocol by the University of Missouri–Kansas City Pediatric IRB. This ancillary data is not shown in full in this report.

### MicroRNA isolation, primers and annotations

Total RNA from 1 mL of serum from each CAMP subject was isolated using Norgen Biotek RNA isolation kit (Thorold, ON, Canada). We averaged 4 ng/ul of total RNA in 20 ul measured by RiboGreen. Isolated RNA was reverse-transcribed and the product was pre-amplified using Megaplex PreAmp Primers and TaqMan PreAmp Master Mix (Applied Biosystems, Grand Island, NY). Total RNA was isolated from human fetal lung tissue samples using the RNeasy mini kit (Qiagen, Valencia, CA).

TaqMan microRNA quantitative PCR primers were from Life Technologies Megaplex RT Primers, Human Pool Set v3.0 (Omaha, NE) which contains 754 primers representing 738 unique human microRNAs (miRBase release 21, https://tools.lifetechnologies.com/content/sfs/brochures/megaplex-pools-array-card-content.xlsx) and 4 housekeeping primers (RNU44, RNU48, U6, ath-MIR159). Samples were run on the QuantStudio 12K Flex Real-Time PCR System with OpenArray Block (Life Technologies, Carlsbad, CA). Initial quality control was performed per manufacturer protocol, using predefined thresholds for amplification scores (>1.24) and Cq (>0.80) confidence intervals. We excluded microRNAs from further analysis if they were detected in fewer than 75% of the 160 subjects (173 samples).

All microRNAs in this study were annotated by their miRBase release 21 (June 2014, ftp://mirbase.org/pub/mirbase/21/)[26] symbol and accession number. The complete dataset is accessible at the NCBI Gene Expression Omnibus (GEO, http://www.ncbi.nih.gov/geo/) as GSE74770.

### Data analysis: Normalization and linear regression model

We quantile normalized (sample-wise) the data matrix of 758 microRNA primers x 173 samples of detected microRNA cycle threshold values (miR_Ct) to the matrix mean using the Matlab (MathWorks Inc, Natick, MA) function *quantilenorm*.

We used miR_Ct values that pass initial quality controls: amplification scores (>1.24) and Cq (>0.80) confidence intervals. We limited our analysis to 108 of the 738 non-housekeeping microRNA primers that were detected in at least 75% of the study samples for their miR_Ct linear associations with the following lung function measures/phenotypes separately: pre-bronchodilator FEV_1_ and FVC as percentages of predicted (FEV_1_% and FVC%) and FEV_1_/FVC, separately [27]. We used least squares linear regression models (Matlab function *regress*) to identify microRNAs with miR_Ct values that are associated with the each phenotype of interest.

For the sample-wise vector FEV_1_/FVC, we used the linear model FEV_1_/FVC = I × B0 + miR_Ct × B1 (**) for each microRNA, where miR_Ct represented the vector of sample-wise quantile-normalized quality-controlled cycle threshold values, I the vector of 1's with the same length as miR_Ct and B1 the scalar regression coefficient of interest. We considered the corresponding microRNA to be significantly associated with FEV_1_/FVC if the 95% confidence interval of B1 did not contain zero. We found 22 microRNAs significantly associated with FEV_1_/FVC. To assess the multiple comparisons error (i.e., false positive rate) in this strategy, we performed a permutation analysis[28] of the miR_Ct data matrix of 108 microRNAs x 173 samples. In each iteration of the permutation analysis, we shuffled the sample FEV_1_/FVC labels once and counted the number of microRNAs that were significant, i.e., whose B1 95% confidence interval excluded zero in (**). We iterated this process up to 10,000 times, each time noting the number of significant microRNAs or false positives whose distribution is shown in Figure A in S1 File. The median and mean number of false positives were 4 and 5.6, corresponding to false discovery rates of 18% (~4/22) and 25% (~5.6/22) respectively. Even though the p-value for the linear regression was not used in our strategy to determine significant association, we show it and the multiple testing adjusted p-value [29] computed using Matlab function *mafdr*.

Besides quantile normalized miR_Ct values, we also considered microRNA-/primer-wise rank normalized miR_Ct and corresponding phenotype variables as model inputs to assess the effect of outlier variables. In order to evaluate the association of the microRNA identified via the initial percent predicted analyses with raw lung function measures, multivariable analyses of FEV_1_ and FVC (in liters), and FEV_1_/FVC were performed, adjusted for age, sex and height, cf. Table B in S1 File.

### Ontology analysis and genome wide associations

Ontological pathways analysis was performed using ClueGO and CluePedia[30, 31] plug-ins in Cytoscape (http://www.cytoscape.org/) focusing on lung function microRNAs that were either previously reported to be associated with asthma or significantly associated with gestational age in our human fetal lung data, i.e., age = I × B0 + miR_Ct × B1. Functionally validated target genes for microRNAs of interest were obtained from miRTarBase (http://mirtarbase.mbc.nctu.edu.tw/ version 15 September 2015) [32]. Genome wide association study (GWAS) linked asthma and lung function mapped ontology traits were obtained from GWAS Catalog, the NHGRI-EBI catalog of published GWAS (https://www.ebi.ac.uk/gwas/ version 18 April 2016).

## Results

### Study population

The population characteristics of the 160 CAMP subjects are shown in Table 1. Due to known effects of race on microRNA expression [23, 24], we limited this study to self-identified non-Hispanic Caucasians. For these subjects, the global characteristics at randomization are representative of the larger CAMP non-Hispanic Caucasian cohort[22] (data not shown).

### Detected circulating microRNAs are involved in asthma susceptibility

Of the 738 non-housekeeping microRNAs annotated with miRBase release 21 on the array, 108 (14.6%) were detected in at least 75% of these pediatric asthma samples, cf. Table A in S1 File. We investigated the tissue specificity and asthma susceptibility of these 108 microRNAs by noting their detection in asthma relative to non-asthma conditions in 15 human tissues studies, Table 2. 74 (68.5%) had evidence of at least 1 reported prior differential expression in relation to human asthma susceptibility, and 34 (31.5%) had been reported in 2 or more studies.

**Table 2:** 15 studies of microRNAs in asthma that were compared with this study. The source tissue and sizes of asthma and control populations are shown.

Author	PubMed ID	Source	# asthma	# control
Jardim, et al. 2012 [33]	22679274	Bronchial epithelia	16	16
Levänen, et al. 2013 [34]	23333113	Bronchoalveolar lavage fluid exosomes	10	10
Liu, et al. 2012 [35]	22895815	Lymphocytes	6	6
Nakano, et al. 2013 [36]	23954351	CD4+ T cells	15	26
Nicodemus-Johnson, et al. 2013 [37]	23534973	Airway epithelia, white blood cells	22 mom with asthma, 33 mom no asthma	0
Panganiban, et al. 2012 [18]	23885321	Serum	10	10
Panganiban, et al. 2016 [19]	27025347	Serum	35	19
Perry, et al. 2014 [38]	23944957	Airway smooth muscles	9 severe, 9 non-severe	9
Pinkerton, et al. 2013 [39]	23628339	Exhaled breath condensate	11	12 normal, 10 COPD
Seumois, et al. 2012 [40]	23304658	CD4+ T cells	6 off ICS, 6 on ICS	10
Solberg, et al. 2012 [41]	22955319	Airway epithelia	16 off ICS, 19 on ICS	12
Suojalehto, et al. 2014 [42]	24513959	Nasal mucosa	117 (54 persistent)	33
Tsitsiou, et al. 2012 [43]	21917308	CD8+ T cells	12 severe, 4 non-severe	8
Williams, et al. 2009 [44]	19521514	Airway biopsies	8 (mild)	8
Yamamoto, et al. 2012 [45]	23170939	Peripheral blood mononuclear cells	7	4

### MicroRNA expression associated with lung function

We investigated the linear association of the microRNA with 3 measures of lung function, FEV_1_/FVC, FEV_1_% and FVC%, cf. Tables 3–5. FEV_1_/FVC had the greatest number of microRNA associations with 22 of the 108 (20.4%) circulating microRNA nominally associated with FEV_1_/FVC with a 18–25% false discovery rate. As indicated in the Materials and Methods section, even though the p-value for the linear regression was not used in our strategy to determine significance, we noted that the p-value and multiple testing adjusted p-value [29] for these genes were <0.05 and <0.25 respectively. 4 microRNAs were differentially expressed in association with FEV_1_%, while 8 were associated with FVC%. Most FEV1% and FEV1/FVC microRNAs were positively associated with lung function outcomes, while most FVC% microRNAs were associated with decrements in this measure of lung function.

**Table 3:** Circulating microRNAs associated with FEV_1_/FVC. The regression slope mean (beta) and its 95% confidence interval are shown. In the second column "Asthma Lit (Fetal)", "Y" indicates a report in at least 1 study listed in Table 2, "Y^2^" indicates a report in 2 or more of these studies, "(f)" indicates significant correlation of the microRNA in fetal lung tissue with gestational age. The third column "Target genes with GWAS asthma/FEV1 change" lists functionally validated target genes from miRTarBase that have GWAS linked asthma and lung function mapped ontology traits in GWAS Catalog.

FEV_1_/FVC	Asthma Lit (fetal)	Target genes with GWAS asthma/FEV1 change	Beta (95% CI)	P-Value
hsa-miR-126-3p	Y (f)	CXCR4, PITPNC1	1.23 (0.56, 1.91)	0.0004**
hsa-miR-1290	Y		0.76 (0.05, 1.48)	0.0372*
hsa-miR-139-5p		CXCR4	1.48 (0.22, 2.74)	0.022*
hsa-miR-142-3p		CCNT2, LRRC32	1.00 (0.06, 1.95)	0.0378*
hsa-miR-146b-5p			1.07 (0.16, 1.98)	0.0209*
hsa-miR-15b-5p	Y		1.98 (0.38, 3.59)	0.016*
hsa-miR-16-5p	Y		0.78 (0.09, 1.47)	0.0269*
hsa-miR-186-5p			1.11 (0.41, 1.82)	0.0022*
hsa-miR-191-5p		CEBPB	1.10 (0.43, 1.76)	0.0014*
hsa-miR-203a-3p	Y^2^ (f)	ASAP1	0.76 (0.01, 1.52)	0.0477*
hsa-miR-206		GPD2, PAX3	-1.15 (-2.12, -0.17)	0.0213*
hsa-miR-26a-5p	Y^2^ (f)		0.99 (0.00, 1.97)	0.0497*
hsa-miR-301a-3p	Y		0.87 (0.02, 1.73)	0.0453*
hsa-miR-30b-5p	Y^2^ (f)		1.30 (0.07, 2.54)	0.0392*
hsa-miR-331-3p		NRP2	1.69 (0.31, 3.06)	0.0166*
hsa-miR-342-3p	Y (f)		0.85 (0.19, 1.5)	0.012*
hsa-miR-374a-5p	Y	CEBPB	1.14 (0.31, 1.97)	0.0076*
hsa-miR-409-3p	(f)		1.18 (0.27, 2.09)	0.0113*
hsa-miR-454-3p	Y^2^		1.13 (0.34, 1.92)	0.0054*
hsa-miR-484			1.54 (0.13, 2.96)	0.0327*
hsa-miR-660-5p	Y		1.61 (0.21, 3.01)	0.0248*
hsa-miR-942-5p	(f)		1.02 (0.01, 2.02)	0.0472*

In the fifth column "P-Value",

** indicates adjusted p-value <0.05,

* indicates adjusted p-value <0.25.

**Table 4:** Circulating microRNAs associated with FEV_1_%. The Table legend is similar to Table 3.

FEV_1_%	Asthma Lit (fetal)	Target genes with GWAS asthma/FEV1 change	Beta (95% CI)	P-Value
hsa-miR-142-3p		CCNT2, LRRC32	2.03 (0.40, 3.66)	0.015
hsa-miR-27b-3p	Y	MMP13, PAX3, PSAP	-2.68 (-5.19, -0.18)	0.036
hsa-miR-374a-5p	Y	CEBPB	1.60 (0.17, 3.03)	0.028
hsa-miR-454-3p	Y^2^		1.37 (0.02, 2.72)	0.046

**Table 5:** Circulating microRNAs associated with FVC%. The Table legend is similar to Table 3.

FVC%	Asthma Lit (fetal)	Target genes with GWAS asthma/FEV1 change	Beta (95% CI)	P-Value
hsa-miR-106b-5p	Y	TWIST1	-2.16 (-4.02, -0.29)	0.024
hsa-miR-15b-5p	Y		-2.76 (-5.34, -0.18)	0.037
hsa-miR-223-5p	Y		-2.11 (-4.21, -0.02)	0.048
hsa-miR-320a	Y^2^ (f)		3.30 (0.34, 6.26)	0.029
hsa-miR-339-3p	Y		-1.66 (-3.21, -0.11)	0.036
hsa-miR-340-5p	Y		-1.62 (-2.95, -0.28)	0.018
hsa-miR-376c-3p			-1.56 (-2.94, -0.18)	0.027
hsa-miR-645			1.75 (0.22, 3.28)	0.026

**Fig 1:**
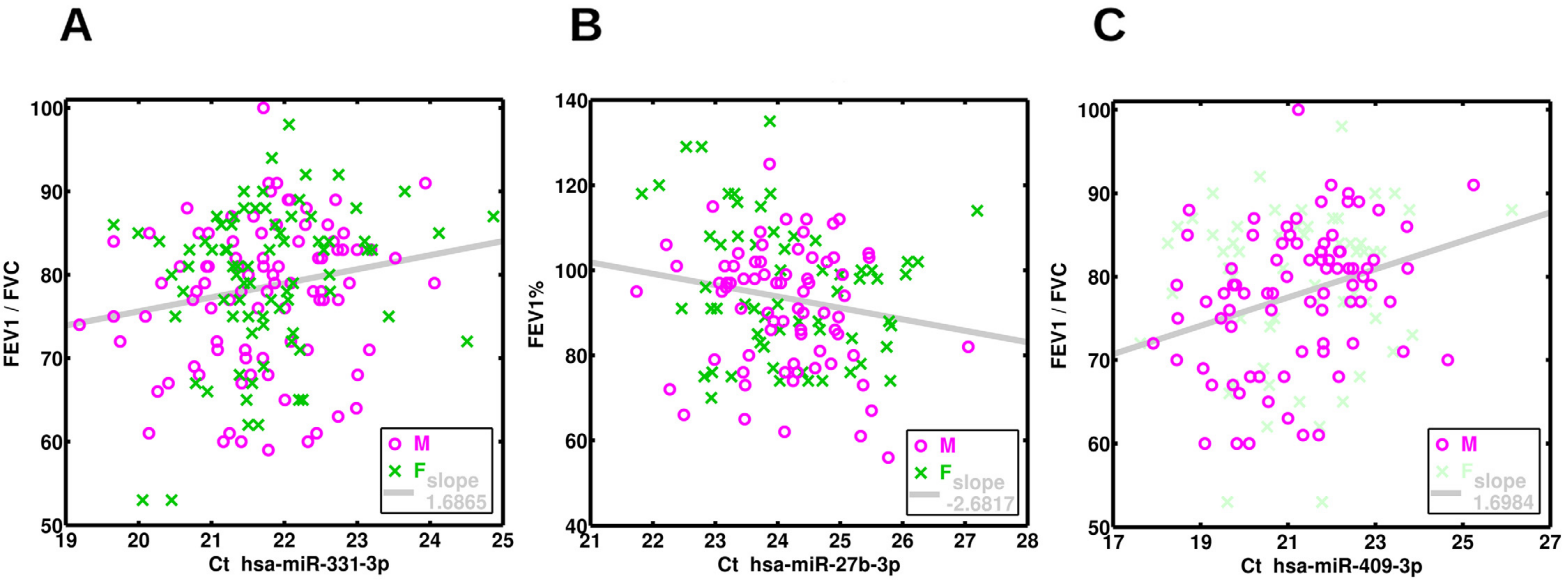
Three examples of microRNAs associated with lung function measures. **(A**) Association of serum miR-331-3p with FEV_1_/FVC in childhood asthma. **(B**) Association of serum miR-27b-3p with FEV_1_% in childhood asthma. **(C**) Sex-specific association of miR-409-3p with FEV_1_/FVC in asthmatic boys.

From an effect estimate perspective, the strongest association with FEV_1_/FVC was miR-15b-5p, which was associated with a 1.98% increase (Beta) in FEV_1_/FVC for every 1 unit Ct change (2-fold change in microRNA expression), likely mediated in part by the strong negative effect of this microRNA on FVC. Similarly, each doubling in miR-27b-3p was associated with an average 2.68% decrease (Beta) in FEV_1_%, and each doubling of miR-320a was associated with an average 3.30% increase (Beta) in FVC%. Three examples of these associations are illustrated in Fig 1. For FEV_1_/FVC, FEV_1_% and FVC% respectively, 12 (55%), 3 (75%) and 6 (75%) of their associated microRNAs had prior evidence of associations with asthma susceptibility in 15 other studies, Table 2. Furthermore, 8 (of 22) FEV_1_/FVC and 3 (of 4) FEV_1_%, and 1 (of 8) FVC% microRNAs had functionally validated target genes that have been linked via GWAS to asthma and FEV1 change, Tables 3–6. All functionally validated target genes of all our FEV_1_/FVC, FEV_1_% and FVC% microRNAs from miRTarBase are listed in Table C in S1 File.

**Table 6:** Asthma and lung function GWAS associations of miRNA target genes. Target genes from Tables 3–5 that have been linked to asthma or FEV1 change in GWAS Catalog.

Target Gene	Entrez ID	Paired microRNA association	Mapped ontology traits on GWAS central
ASAP1	50807	FEV1/FVC	asthma, FEV change measurement, response to bronchodilator, response to glucocorticoid
CCNT2	905	FEV1/FVC, FEV1%	asthma, FEV change measurement, response to bronchodilator, response to glucocorticoid
CXCR4	7852	FEV1/FVC	asthma, FEV change measurement, response to bronchodilator
GPD2	2820	FEV1/FVC	asthma, FEV change measurement, response to bronchodilator, response to corticosteroid
LRRC32	2615	FEV1/FVC, FEV1%	asthma, FEV change measurement, response to bronchodilator, response to glucocorticoid
MMP13	4322	FEV1%	asthma, FEV change measurement, response to bronchodilator
NRP2	8828	FEV1/FVC	asthma, FEV change measurement, response to bronchodilator, response to glucocorticoid
PAX3	5077	FEV1/FVC, FEV1%	asthma, FEV change measurement, response to bronchodilator, response to glucocorticoid
PITPNC1	26207	FEV1/FVC	asthma, FEV change measurement, response to bronchodilator, response to glucocorticoid
PSAP	5660	FEV1%	asthma, FEV change measurement, FEV/FEC ratio, pulmonary function measurement, response to bronchodilator
TWIST1	7291	FVC%	asthma, FEV/FEC ratio, forced expiratory volume

### Sex specific microRNA associations with FEV_1_/FVC

Given the preponderance of lung function associations with FEV_1_/FVC, and the known differences in the ratio by sex, we hypothesized that sex-specific differences in lung development may play a role. We investigate this by analyzing the 22 FEV_1_/FVC associated microRNAs above via sex stratified analyses. 9 (40.9%) of the 22 microRNA were associated in males alone, with 3 (13.6%) associated in females alone, Table 7. miR-409-3p is an example of a male associated FEV_1_/FVC microRNA, Fig 1C.

**Table 7:** Sex stratified linear associations for the 22 FEV_1_/FVC microRNAs with the regression slope mean (beta) and its 95% confidence interval. In the first column, "(f)" indicates significant correlation of the microRNA in fetal lung tissue with gestational age.

FEV_1_/FVC ^(fetal)^	Male Association	Male P-Value	Female Association	Female P-Value
hsa-miR-126-3p ^(f)^	Yes	0.045	Yes	0.004
hsa-miR-1290	No	0.889	Yes	0.003
hsa-miR-139-5p	Yes	0.048	No	0.186
hsa-miR-142-3p	No	0.577	Yes	0.028
hsa-miR-146b-5p	No	0.114	No	0.095
hsa-miR-15b-5p	Yes	0.028	No	0.222
hsa-miR-16-5p	No	0.257	No	0.057
hsa-miR-186-5p	Yes	0.019	No	0.058
hsa-miR-191-5p	No	0.142	Yes	0.002
hsa-miR-203a-3p ^(f)^	No	0.338	No	0.066
hsa-miR-206	No	0.056	No	0.338
hsa-miR-26a-5p ^(f)^	No	0.138	No	0.207
hsa-miR-301a-3p	No	0.251	No	0.084
hsa-miR-30b-5p ^(f)^	No	0.258	No	0.087
hsa-miR-331-3p	No	0.134	No	0.067
hsa-miR-342-3p ^(f)^	Yes	0.044	No	0.132
hsa-miR-374a-5p	Yes	0.032	No	0.106
hsa-miR-409-3p ^(f)^	Yes	0.005	No	0.415
hsa-miR-454-3p	Yes	0.028	No	0.081
hsa-miR-484	No	0.143	No	0.132
hsa-miR-660-5p	Yes	0.032	No	0.339
hsa-miR-942-5p ^(f)^	Yes	0.041	No	0.465

### Lung function microRNAs in fetal lung development

In order to elucidate the role that developmental alterations might play in the microRNA-lung function associations, we analyzed the 22 FEV_1_/FVC microRNAs for association with developmental gestational age in a sample of 30 human fetal lung samples; we have previously described the utility of these samples in a transcriptomic study of human lung development[46]. 7 (31.8%) of the 22 microRNAs were associated with gestational age: miR-126-3p, miR-203a-3p, miR-26a-5p, miR-30b-5p, miR-342-3p, miR-409-3p and miR-942-5p, Table 3. This included 3 (miR-342-3p, miR-409-3p and miR-942-5p) associated with the male sex-specific FEV_1_/FVC analysis, Table 7.

### Ontology analyses of lung function microRNAs

**Fig 2:**
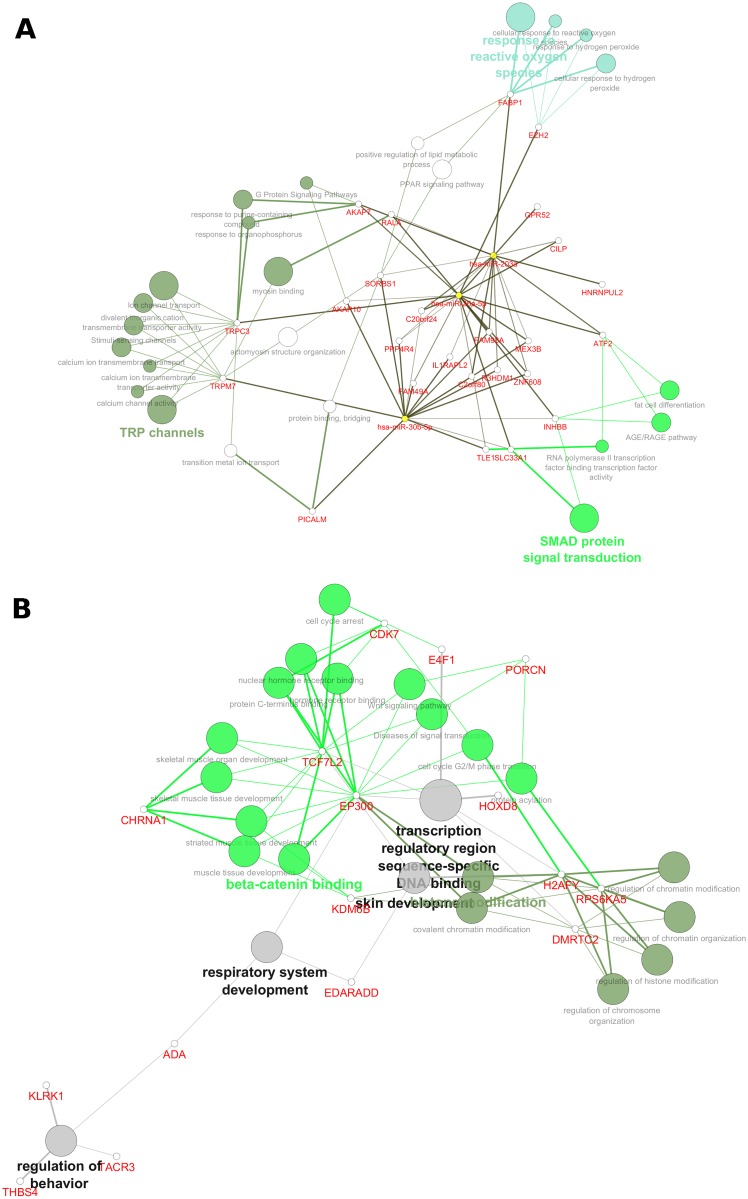
Ontology analyses of FEV_1_/FVC microRNAs that were associated with other asthma microRNA studies or fetal lung development. **(A**) FEV_1_/FVC microRNAs (miR-203a-3p, miR-26a-5p, miR-30b-5p and miR-454-3p) that were reported in at least 2 of 15 other asthma case-control studies listed in Table 2. **(B**) FEV_1_/FVC (miR-342-3p, miR-409-3p and miR-942-5p) microRNAs that were associated with boys alone in the sex stratified analysis and also correlated with gestational age in our fetal lung samples.

Pathway analyses focusing two sets of overlapping microRNA associations were performed using ClueGO and CluePedia[30, 31] plug-ins in Cytoscape. The first analysis focused on the FEV_1_/FVC microRNAs that were previously reported as differentially expressed in at least 2 case-control asthma studies, Table 3: miR-203a-3p, miR-26a-5p, miR-30b-5p and miR-454-3p. These 4 microRNAs were assessed for common target connectivity and associated pathways, Fig 2A. Multiple pathways are represented by these targets including PPAR signaling, G-protein coupled signaling, actin and myosin binding, fat cell differentiation, and SMAD protein signaling.

Our second pathway analysis focused on the 3 FEV_1_/FVC microRNAs that were associated with boys alone in the sex stratified analysis and also correlated with gestational age in our fetal lung samples: miR-342-3p, miR-409-3p and miR-942-5p. The significant pathways here included chromatin and histone modification, respiratory system development and the β-catenin pathway, Fig 2B.

## Discussion

Circulating microRNAs offer the potential to capture the inflammatory milieu in large-scale population studies without the expense and invasiveness required for direct studies of asthmatic airway cells. Since serum is easily measured, microRNAs may serve as disease biomarkers. In this study, we interrogated serum samples from 160 childhood asthmatics upon entry into a large clinical trial. Following quality control, 108 microRNAs were expressed in at least 75% of the samples. Of these, 74 (68.5%) had previously been associated with asthma susceptibility in at least one prior study of airway cells, supporting the easy detection of disease specific microRNAs in circulation. As measures of lung function, we interrogated the role of circulating microRNAs in relation to FEV_1_%, FVC% and FEV_1_/FVC; noting that a significant proportion of the detected circulating microRNAs were associated with FEV_1_/FVC and a smaller number with FEV_1_% and FVC% (Tables 3–5). These associations included microRNAs both previously associated with asthma, as well as a significant proportion of novel associations. 8 (of 22) FEV_1_/FVC and 3 (of 4) FEV_1_%, and 1 (of 8) FVC% microRNAs had functionally validated target genes that have been linked via GWAS to asthma and FEV1 change, Together, these data support the biological role of microRNAs in asthma severity, and the easily measurable microRNAs as biomarkers of lung function within the circulation of asthmatics.

Of the 22 microRNAs associated with FEV_1_/FVC, 12 had previously been associated with asthma, Table 3: miR-126-3p, miR-1290, miR-15b-5p, miR-16-5p, miR-203a-3p, miR-26a-5p, miR-30b-5p, miR-301a-3p, miR-342-3p, miR-374a-5p, miR-454-3p and miR-660-5p. Of these, 4 microRNAs (miR-203a-3p, miR-26a-5p, miR-30b-5p and miR-454-3p) were previously reported as differentially expressed in at least 2 case-control asthma studies, with each associated in at least one study of bronchoscopically sampled airway cells. This supports the hypothesis that microRNAs may influence asthma susceptibility directly via targeting genes and cellular processes that may be involved in augmenting airflow obstruction. For instance, miR-203 has been associated with asthma in two airway epithelial cell studies[33, 41] and is upregulated in the serum of subjects with atopic dermatitis[47]. Several studies have highlighted the potential of miR-203 to influence asthma via inflammatory mechanisms, which may result in the abnormalities in FEV_1_/FVC noted in the current study. These include the association of increases in serum miR-203 with increased IgE level[47] and with airway epithelial cell apoptosis[48]. miR-26a has previously been associated with asthma in studies of bronchial epithelial cells, bronchoalveolar lavage fluid and serum[18, 34, 41]. In contrast to the inflammatory effects of miR-203, we have reported that miR-26a is strongly expressed in airway smooth muscle (ASM) cells [49]. Prior work has indicated that miR-26a is induced in human ASM cells following physical stretch and that increased levels of miR-26a causes ASM cellular hypertrophy by directly targeting *GSK3B* (glycogen synthase kinase-3b)[50]. This effect is reversible following miR-26a inhibition[50]. While direct molecular mechanistic functional experiments are beyond the scope of the current study, we emphasize that these associations are consistent with divergent biologic effects on differing airway cells affecting lung function via microRNAs and that these airway phenomena are easily detectable via serum sampling.

While known asthma microRNA associations with lung function in asthma support direct biologic modulation resulting from disease susceptibility, novel microRNAs were also associated with FEV_1_/FVC. For instance miR-186-5p, while not previously associated with asthma, may play a crucial role in the regulation of acetylcholine packaging and degradation [51]. In turn, the addition of anticholinergic therapy in patients with moderate to severe asthma has been shown to significantly improve lung function [52]. Thus, miR-186-5p may influence lung function via the modulation of airway tone via the cholinergic pathway.

Of the 4 microRNAs associated with FEV_1_%, 3 (miR-142-3p, miR-374-5p and miR-454-3p) were also associated with FEV_1_/FVC, supporting a common underlying mechanism. Both miR-374 and miR-454 have previously been associated with asthma in a case-control study of bronchial epithelial cells[33]. While little is known about the effect of these microRNAs on the airway, miR-374 target genes are enriched in alveolar epithelial cells during hyperoxic stress and recovery supporting a role in structural repair of the lung[53]. Only one microRNA, miR-15b-5p was noted in common with both FVC% and FEV_1_/FVC. miR-15b is down-regulated in association with asthma[36]. While the mechanistic basis for this association is unknown, lower lung miR-15b also differentiates smokers with chronic obstructive pulmonary disease from smokers without airflow obstruction via altered TGFβ signaling, supporting this as a potential role for this microRNA in lung function[54].

While the reported circulating microRNAs may directly influence lung function in asthma via active targeting of inflammatory or structural biology relevant to asthma, microRNAs are also key drivers of normal human embryonic and fetal development [55, 56], including that of the lung[57, 58]. Disruptions in the FEV_1_/FVC may arise as a result of dysanapsis [59], nonisotropic growth of lung airways and parenchyma is a feature of asthma and airway hyperresponsiveness which is greater in early childhood in boys than girls. We therefore tested for potential dysanapsis via analyses stratified by sex. Of the 22 circulating microRNAs associated with FEV_1_/FVC, over half of the microRNAs (9 in boys and 3 in girls) were significantly associated in one sex only, Table 7. To further explore whether these microRNAs might be mediators of lung developmental processes, we then examined these microRNAs for association with developmental age in a collection of human fetal lungs[46]. Globally, 7 of the 22 FEV_1_/FVC microRNAs were also associated with gestational age, with 3 (miR-342-3p, miR-409-3p and miR-942-5p) associated with both human fetal lung development and with FEV_1_/FVC in asthmatic boys alone. The developmental role of miR-409-3p has previously been postulated in non-asthmatic lung disease; miR-409 is differentially expressed in fetal lungs and in patients with idiopathic pulmonary fibrosis[60]. The developmental association of miR-342-3p is also intriguing, as it encodes for adipocyte differentiation from mesenchymal stem cells[61, 62]. While this may not directly affect FEV_1_/FVC in asthma, we have previously reported that increases in body mass index (BMI) are associated with decrements in FEV_1_/FVC in the CAMP cohort; these associations are significantly stronger in boys than girls[63]. Thus, developmental alterations regulated via microRNAs may directly influence airway biology through influences on the developing lung or indirectly alter lung function via other mechanisms.

Pathway analyses of the predicted microRNA targets from our analyses are consistent with the mediation of important asthma and developmental pathways via microRNAs resulting in altered lung function. For instance, in the evaluation of the 3 microRNAs associated with both lung function and consistently aligned with asthma susceptibility (Fig 2), multiple pathways were over-represented, including PPAR signaling, G-protein coupled signaling, and myosin binding. The β_2_-adrenergic receptor is a prime example of a G-protein coupled receptor and its role in airway tone and asthma has been widely espoused[64]; we have previously reported the genetic association of haplotypes within the β_2_AR gene with FEV_1_/FVC[65]. Peroxisome proliferator-activated receptor gamma (PPARG) is augmented in the bronchial submucosa, the airway epithelium, and the smooth muscle of asthmatics, as compared with control subjects. The intensity of PPARG expression in bronchial submucosa, as well as airway epithelium and smooth muscle, is negatively related to FEV_1_ [66]. Therapeutic targeting of the PPAR pathway has been postulated for treatment of a variety of inflammatory lung diseases, including asthma[67]. Finally, myosin-binding mediates airway smooth muscle tone and has long been implicated in dynamic airway luminal narrowing and airways responsiveness in asthma[68, 69].

Similarly, the microRNA targets of resulting from the overlap analysis of microRNAs associated with both fetal lung development and lung function represented pathways carefully aligned with significant biology, including respiratory system development and the β-catenin pathway. β-catenin not only regulates cell to cell adhesion as a protein interacting with cadherin, but also functions as a component of the Wnt signaling pathway[70]. In turn, the Wnt/β-catenin pathway is crucial for the patterning of the early lung morphogenesis in mice and humans[71]. We have previously implicated genotypic variation within the Wnt signaling pathway with alterations in lung function in childhood asthmatics[25]. Recently, it has been shown that modulation of the β-catenin pathway can abrogate experimental models of allergic airways disease[72], lending further potential significance to our findings.

Our study has a number of unique strengths. Among these are a large sample size combined with a large number of interrogated microRNAs. Our sample size provides us with the power to detect associations despite lower starting concentrations of microRNAs within the circulation. The CAMP cohort was well characterized using standardized lung function methodologies and blood sampling procedures across the CAMP clinical sites, thereby minimizing bias related to measurement error. Additionally, we performed analysis of biologic replicates in about 10% of the population cohort that showed high microRNA-microRNA correlations (rank correlations of >0.90 for the replicate samples—data not shown). Among the weaknesses to this study include the fact that the reported associations have yet to be functionally validated. We note that the CAMP clinical trial randomization occurred approximately 20 years ago[21, 22]. Despite this, multiple studies have shown that carefully stored samples can yield reliable microRNA concentrations months to years later[7, 8, 73]. That we are able to detect a substantial number of asthma associated microRNAs within the serum of the CAMP asthmatics 20 years following randomization enhances the validity of our approach. Finally, we note that 74 microRNAs previously associated with asthma were well detected in our serum samples; the majority of these were not associated in this study of lung function in asthmatic children. It is likely that these microRNAs function to influence asthma in ways independent of structural airways biology, such as via the immune or inflammatory response. Additional studies of these circulating microRNAs in conjunction with other phenotypes are warranted.

In conclusion, serum microRNAs are associated with lung function measures in the asthmatic child and appear to reflect in vivo airway cell biology. These findings support the likelihood that microRNAs can serve as easily measurable, circulating biomarkers of asthma severity. Given that microRNAs have been shown to have prognostic value in both cancer[9, 74–77] and other inflammatory processes[78–82], futher studies on the predictive value of microRNAs in relation to asthma outcomes are warranted.

## Supporting Information

S1 File This file contains Figure A, and Tables A-C described below. The distribution of the number of false positives or significant microRNAs from 10,000 permutations of the sample FEV1/FVC labels of the 108 microRNAs x 173 samples data matrix (Figure A). 108 microRNAs identified by their miRBase release 21 symbol measured in our Childhood Asthma Management Program (CAMP) serum samples: 160 subjects, 173 samples. Asthma Literature (Column 2) is based on detection in 15 asthma microRNA studies in Table 2. Yes indicates report in at least 1 study. Yes^2^ indicates report in 2 or more studies (Table A). Multivariable microRNA-lung function associations adjusted for age, sex and height. FEV1 and FVC values in liters (Table B). All functionally validated target genes for our FEV_1_/FVC, FEV_1_% and FVC% microRNAs from miRTarBase (http://mirtarbase.mbc.nctu.edu.tw/ version 15 September 2015) (Table C).
(DOC)
