# Supplementary material for: Circulating MicroRNAs: Association with Lung Function in Asthma
Source: PLoS One. 2016 Jun 30;11(6):e0157998. doi: 10.1371/journal.pone.0157998 (PMC4928864; doi:10.1371/journal.pone.0157998)
Supplement: S1 File — The distribution of the number of false positives or significant microRNAs from 10,000 permutations of the sample FEV1/FVC labels of the 108 microRNAs x 173 samples data matrix (Figure A). 108 microRNAs identified by their miRBase release 21 symbol measured in our Childhood Asthma Management Program (CAMP) serum samples: 160 subjects, 173 samples. Asthma Literature (Column 2) is based on detection in 15 asthma microRNA studies in Table 2. Yes indicates report in at least 1 study. Yes2 indicates report in 2 or more studies (Table A). Multivariable microRNA-lung function associations adjusted for age, sex and height. FEV1 and FVC values in liters (Table B). All functionally validated target genes for our FEV1/FVC, FEV1% and FVC% microRNAs from miRTarBase (http://mirtarbase.mbc.nctu.edu.tw/ version 15 September 2015) (Table C). (DOC) [file pone.0157998.s001.doc]

**SUPPORTING INFORMATION**

**Figure A. The distribution of the number of false positives or significant microRNAs from 10,000 permutations of the sample FEV1/FVC labels of the 108 microRNAs x 173 samples data matrix**.

**
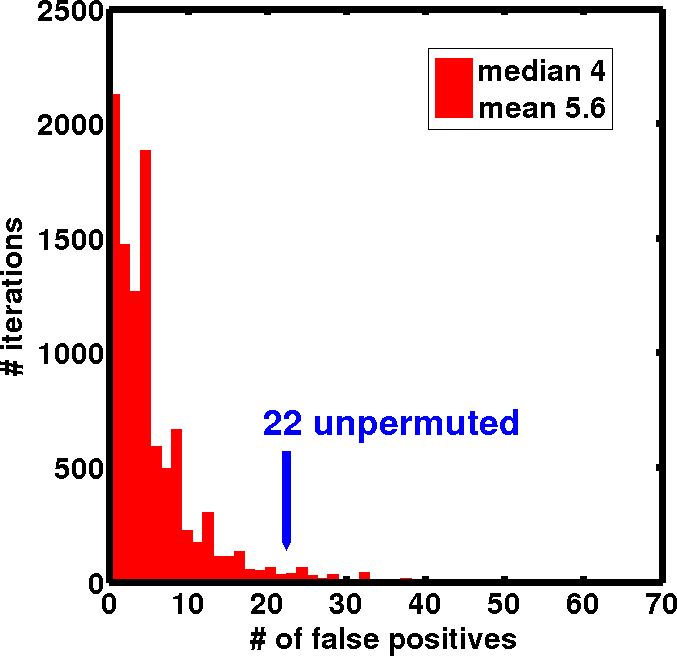
**

**Table A. 108 microRNAs identified by their miRBase release 21 symbol measured in our Childhood Asthma Management Program (CAMP) serum samples: 160 subjects, 173 samples.** Asthma Literature (Column 2) is based on detection in 15 asthma microRNA studies in **Table 6**. Yes indicates report in at least 1 study. Yes^2^ indicates report in 2 or more studies.

| **microRNA** | **Asthma Literature** | **# Samples** | **Assay ID** | **Sequence** |
| --- | --- | --- | --- | --- |
| hsa-let-7c-5p | Yes^2^ | 158 | 000379 | UGAGGUAGUAGGUUGUAUGGUU |
| hsa-let-7d-5p | Yes^2^ | 131 | 002283 | AGAGGUAGUAGGUUGCAUAGUU |
| hsa-miR-101-3p | Yes | 140 | 002253 | UACAGUACUGUGAUAACUGAA |
| hsa-miR-106a-5p | Yes^2^ | 173 | 002169 | AAAAGUGCUUACAGUGCAGGUAG |
| hsa-miR-106b-5p | Yes | 168 | 000442 | UAAAGUGCUGACAGUGCAGAU |
| hsa-miR-10a-5p | Yes | 130 | 000387 | UACCCUGUAGAUCCGAAUUUGUG |
| hsa-miR-10b-3p |  | 169 | 002315 | ACAGAUUCGAUUCUAGGGGAAU |
| hsa-miR-122-5p |  | 134 | 002245 | UGGAGUGUGACAAUGGUGUUUG |
| hsa-miR-126-5p | Yes | 161 | 000451 | CAUUAUUACUUUUGGUACGCG |
| hsa-miR-126-3p | Yes | 171 | 002228 | UCGUACCGUGAGUAAUAAUGCG |
| hsa-miR-127-3p | Yes | 149 | 000452 | UCGGAUCCGUCUGAGCUUGGCU |
| hsa-miR-1290 | Yes | 160 | 002863 | UGGAUUUUUGGAUCAGGGA |
| hsa-miR-1291 | Yes^2^ | 148 | 002838 | UGGCCCUGACUGAAGACCAGCAGU |
| hsa-miR-130a-3p | Yes^2^ | 166 | 000454 | CAGUGCAAUGUUAAAAGGGCAU |
| hsa-miR-130b-3p | Yes | 135 | 000456 | CAGUGCAAUGAUGAAAGGGCAU |
| hsa-miR-138-5p | Yes^2^ | 125 | 002284 | AGCUGGUGUUGUGAAUCAGGCCG |
| hsa-miR-139-5p |  | 157 | 002289 | UCUACAGUGCACGUGUCUCCAG |
| hsa-miR-140-3p | Yes^2^ | 133 | 002234 | UACCACAGGGUAGAACCACGG |
| hsa-miR-142-3p |  | 170 | 000464 | UGUAGUGUUUCCUACUUUAUGGA |
| hsa-miR-144-5p |  | 130 | 002148 | GGAUAUCAUCAUAUACUGUAAG |
| hsa-miR-146a-5p | Yes | 163 | 000468 | UGAGAACUGAAUUCCAUGGGUU |
| hsa-miR-146b-5p |  | 166 | 001097 | UGAGAACUGAAUUCCAUAGGCU |
| hsa-miR-148a-3p | Yes^2^ | 123 | 000470 | UCAGUGCACUACAGAACUUUGU |
| hsa-miR-150-5p | Yes^2^ | 160 | 000473 | UCUCCCAACCCUUGUACCAGUG |
| hsa-miR-152-3p |  | 167 | 000475 | UCAGUGCAUGACAGAACUUGG |
| hsa-miR-15b-5p | Yes | 137 | 000390 | UAGCAGCACAUCAUGGUUUACA |
| hsa-miR-16-5p | Yes^2^ | 172 | 000391 | UAGCAGCACGUAAAUAUUGGCG |
| hsa-miR-17-5p | Yes | 172 | 002308 | CAAAGUGCUUACAGUGCAGGUAG |
| hsa-miR-181a-5p | Yes^2^ | 156 | 000480 | AACAUUCAACGCUGUCGGUGAGU |
| hsa-miR-185-5p | Yes | 134 | 002271 | UGGAGAGAAAGGCAGUUCCUGA |
| hsa-miR-186-5p |  | 171 | 002285 | CAAAGAAUUCUCCUUUUGGGCU |
| hsa-miR-191-5p |  | 173 | 002299 | CAACGGAAUCCCAAAAGCAGCUG |
| hsa-miR-192-5p | Yes^2^ | 158 | 000491 | CUGACCUAUGAAUUGACAGCC |
| hsa-miR-193a-5p | Yes^2^ | 158 | 002281 | UGGGUCUUUGCGGGCGAGAUGA |
| hsa-miR-193b-3p | Yes | 142 | 002367 | AACUGGCCCUCAAAGUCCCGCU |
| hsa-miR-195-5p |  | 157 | 000494 | UAGCAGCACAGAAAUAUUGGC |
| hsa-miR-199a-3p |  | 126 | 002304 | ACAGUAGUCUGCACAUUGGUUA |
| hsa-miR-19a-3p | Yes | 163 | 000395 | UGUGCAAAUCUAUGCAAAACUGA |
| hsa-miR-19b-3p | Yes^2^ | 171 | 000396 | UGUGCAAAUCCAUGCAAAACUGA |
| hsa-miR-203a-3p | Yes^2^ | 153 | 000507 | GUGAAAUGUUUAGGACCACUAG |
| hsa-miR-204-5p |  | 125 | 000508 | UUCCCUUUGUCAUCCUAUGCCU |
| hsa-miR-206 |  | 153 | 000510 | UGGAAUGUAAGGAAGUGUGUGG |
| hsa-miR-20a-5p | Yes^2^ | 165 | 000580 | UAAAGUGCUUAUAGUGCAGGUAG |
| hsa-miR-20b-5p | Yes | 150 | 001014 | CAAAGUGCUCAUAGUGCAGGUAG |
| hsa-miR-21-5p | Yes^2^ | 160 | 000397 | UAGCUUAUCAGACUGAUGUUGA |
| hsa-miR-212-3p |  | 128 | 000515 | UAACAGUCUCCAGUCACGGCC |
| hsa-miR-215-5p |  | 126 | 000518 | AUGACCUAUGAAUUGACAGAC |
| hsa-miR-218-5p | Yes | 153 | 000521 | UUGUGCUUGAUCUAACCAUGU |
| hsa-miR-221-3p | Yes | 143 | 000524 | AGCUACAUUGUCUGCUGGGUUUC |
| hsa-miR-222-3p | Yes^2^ | 150 | 002276 | AGCUACAUCUGGCUACUGGGU |
| hsa-miR-223-5p | Yes | 145 | 002098 | CGUGUAUUUGACAAGCUGAGUU |
| hsa-miR-223-3p | Yes^2^ | 172 | 002295 | UGUCAGUUUGUCAAAUACCCCA |
| hsa-miR-24-3p | Yes^2^ | 170 | 000402 | UGGCUCAGUUCAGCAGGAACAG |
| hsa-miR-25-3p |  | 171 | 000403 | CAUUGCACUUGUCUCGGUCUGA |
| hsa-miR-26a-5p | Yes^2^ | 164 | 000405 | UUCAAGUAAUCCAGGAUAGGCU |
| hsa-miR-26b-5p | Yes^2^ | 165 | 000407 | UUCAAGUAAUUCAGGAUAGGU |
| hsa-miR-27a-3p | Yes^2^ | 168 | 000408 | UUCACAGUGGCUAAGUUCCGC |
| hsa-miR-27b-3p | Yes | 134 | 000409 | UUCACAGUGGCUAAGUUCUGC |
| hsa-miR-28-5p |  | 161 | 000411 | AAGGAGCUCACAGUCUAUUGAG |
| hsa-miR-296-5p |  | 140 | 000527 | AGGGCCCCCCCUCAAUCCUGU |
| hsa-miR-29a-3p | Yes^2^ | 158 | 002112 | UAGCACCAUCUGAAAUCGGUUA |
| hsa-miR-29c-3p | Yes | 123 | 000587 | UAGCACCAUUUGAAAUCGGUUA |
| hsa-miR-301a-3p | Yes | 155 | 000528 | CAGUGCAAUAGUAUUGUCAAAGC |
| hsa-miR-30a-5p | Yes^2^ | 162 | 000417 | UGUAAACAUCCUCGACUGGAAG |
| hsa-miR-30b-5p | Yes^2^ | 158 | 000602 | UGUAAACAUCCUACACUCAGCU |
| hsa-miR-30c-5p | Yes^2^ | 158 | 000419 | UGUAAACAUCCUACACUCUCAGC |
| hsa-miR-30d-5p | Yes^2^ | 171 | 000420 | UGUAAACAUCCCCGACUGGAAG |
| hsa-miR-30e-3p | Yes | 140 | 000422 | CUUUCAGUCGGAUGUUUACAGC |
| hsa-miR-320a | Yes^2^ | 172 | 002277 | AAAAGCUGGGUUGAGAGGGCGA |
| hsa-miR-320b | Yes | 138 | 002844 | AAAAGCUGGGUUGAGAGGGCAA |
| hsa-miR-324-5p | Yes | 138 | 000539 | CGCAUCCCCUAGGGCAUUGGUGU |
| hsa-miR-328-3p | Yes | 171 | 000543 | CUGGCCCUCUCUGCCCUUCCGU |
| hsa-miR-331-3p |  | 171 | 000545 | GCCCCUGGGCCUAUCCUAGAA |
| hsa-miR-335-5p | Yes^2^ | 153 | 000546 | UCAAGAGCAAUAACGAAAAAUGU |
| hsa-miR-339-3p | Yes | 142 | 002184 | UGAGCGCCUCGACGACAGAGCCG |
| hsa-miR-340-5p | Yes | 131 | 002258 | UUAUAAAGCAAUGAGACUGAUU |
| hsa-miR-340-3p | Yes | 135 | 002259 | UCCGUCUCAGUUACUUUAUAGC |
| hsa-miR-342-3p | Yes | 158 | 002260 | UCUCACACAGAAAUCGCACCCGU |
| hsa-miR-345-5p | Yes | 163 | 002186 | GCUGACUCCUAGUCCAGGGCUC |
| hsa-miR-374a-5p | Yes | 172 | 000563 | UUAUAAUACAACCUGAUAAGUG |
| hsa-miR-375 | Yes | 166 | 000564 | UUUGUUCGUUCGGCUCGCGUGA |
| hsa-miR-376a-3p |  | 156 | 000565 | AUCAUAGAGGAAAAUCCACGU |
| hsa-miR-376c-3p |  | 152 | 002122 | AACAUAGAGGAAAUUCCACGU |
| hsa-miR-409-3p |  | 143 | 002332 | GAAUGUUGCUCGGUGAACCCCU |
| hsa-miR-410-3p |  | 144 | 001274 | AAUAUAACACAGAUGGCCUGU |
| hsa-miR-411-5p | Yes | 128 | 001610 | UAGUAGACCGUAUAGCGUACG |
| hsa-miR-423-5p | Yes^2^ | 120 | 002340 | UGAGGGGCAGAGAGCGAGACUUU |
| hsa-miR-454-3p | Yes^2^ | 158 | 002323 | UAGUGCAAUAUUGCUUAUAGGGU |
| hsa-miR-483-5p | Yes^2^ | 160 | 002338 | AAGACGGGAGGAAAGAAGGGAG |
| hsa-miR-484 |  | 157 | 001821 | UCAGGCUCAGUCCCCUCCCGAU |
| hsa-miR-539-5p |  | 122 | 001286 | GGAGAAAUUAUCCUUGGUGUGU |
| hsa-miR-548a-3p |  | 153 | 001538 | CAAAACUGGCAAUUACUUUUGC |
| hsa-miR-548c-3p |  | 142 | 001590 | CAAAAAUCUCAAUUACUUUUGC |
| hsa-miR-574-3p | Yes | 130 | 002349 | CACGCUCAUGCACACACCCACA |
| hsa-miR-590-5p | Yes | 155 | 001984 | GAGCUUAUUCAUAAAAGUGCAG |
| hsa-miR-601 |  | 141 | 001558 | UGGUCUAGGAUUGUUGGAGGAG |
| hsa-miR-645 |  | 139 | 001597 | UCUAGGCUGGUACUGCUGA |
| hsa-miR-659-3p |  | 140 | 001514 | CUUGGUUCAGGGAGGGUCCCCA |
| hsa-miR-660-5p | Yes | 152 | 001515 | UACCCAUUGCAUAUCGGAGUUG |
| hsa-miR-744-5p | Yes | 148 | 002324 | UGCGGGGCUAGGGCUAACAGCA |
| hsa-miR-885-5p | Yes | 159 | 002296 | UCCAUUACACUACCCUGCCUCU |
| hsa-miR-92a-3p | Yes | 165 | 000431 | UAUUGCACUUGUCCCGGCCUGU |
| hsa-miR-942-5p |  | 161 | 002187 | UCUUCUCUGUUUUGGCCAUGUG |
| hsa-miR-99b-5p |  | 169 | 000436 | CACCCGUAGAACCGACCUUGCG |
| hsa-miR-140-5p | Yes^2^ | 165 | 001187 | CAGUGGUUUUACCCUAUGGUAG |
| hsa-miR-374b-5p |  | 124 | 001319 | AUAUAAUACAACCUGCUAAGUG |
| hsa-miR-451a |  | 165 | 001141 | AAACCGUUACCAUUACUGAGUU |
| hsa-miR-93-5p | Yes | 160 | 001090 | CAAAGUGCUGUUCGUGCAGGUAG |

**Table B. Multivariable microRNA-lung function associations adjusted for age, sex and height. FEV1 and FVC values in liters.**

| **FEV_1_/FVC** | **Beta (95% CI)** |
| --- | --- |
| hsa-miR-126-3p | 1.12 (0.46, 1.77) |
| hsa-miR-1290 | 0.51 (-0.18, 1.19) |
| hsa-miR-139-5p | 1.37 (0.17, 2.56) |
| hsa-miR-142-3p | 0.69 (-0.22, 1.59) |
| hsa-miR-146b-5p | 1.05 (0.19, 1.91) |
| hsa-miR-15b-5p | 1.99 (0.44, 3.53) |
| hsa-miR-16-5p | 0.74 (0.08, 1.41) |
| hsa-miR-186-5p | 1.02 (0.35, 1.68) |
| hsa-miR-191-5p | 0.96 (0.32, 1.6) |
| hsa-miR-203a-3p | 0.59 (-0.14, 1.32) |
| hsa-miR-206 | -0.85 (-1.79, 0.08) |
| hsa-miR-26a-5p | 0.76 (-0.17, 1.69) |
| hsa-miR-301a-3p | 0.77 (-0.04, 1.59) |
| hsa-miR-30b-5p | 0.89 (-0.31, 2.09) |
| hsa-miR-331-3p | 1.30 (0, 2.6) |
| hsa-miR-342-3p | 0.85 (0.22, 1.48) |
| hsa-miR-374a-5p | 0.89 (0.1, 1.68) |
| hsa-miR-409-3p | 1.20 (0.35, 2.05) |
| hsa-miR-454-3p | 1.00 (0.25, 1.76) |
| hsa-miR-484 | 1.48 (0.09, 2.87) |
| hsa-miR-660-5p | 1.54 (0.23, 2.84) |
| hsa-miR-942-5p | 0.96 (0.03, 1.89) |
|  |  |
| **FEV_1_** | **Beta (95% CI)** |
| hsa-miR-142-3p | 0.024 (0, 0.05) |
| hsa-miR-27b-3p | -0.042 (-0.08, 0) |
| hsa-miR-374a-5p | 0.02 (0, 0.04) |
| hsa-miR-454-3p | 0.028 (0.01, 0.05) |
|  |  |
| **FVC** | **Beta (95% CI)** |
| hsa-miR-106b-5p | -0.02 (-0.06, 0.01) |
| hsa-miR-15b-5p | -0.06 (-0.11, -0.01) |
| hsa-miR-223-5p | -0.04 (-0.08, 0) |
| hsa-miR-320a | 0.08 (0.03, 0.14) |
| hsa-miR-339-3p | -0.03 (-0.06, 0) |
| hsa-miR-340-5p | -0.02 (-0.05, 0) |
| hsa-miR-376c-3p | -0.03 (-0.05, 0) |
| hsa-miR-645 | 0.03 (0, 0.06) |

**Table C. All functionally validated target genes for our FEV_1_/FVC, FEV_1_% and FVC% microRNAs from miRTarBase (http://mirtarbase.mbc.nctu.edu.tw/ version 15 September 2015).**

| **microRNA** | **Target Gene Symbol** | **Target Entrez ID** | **GWAS asthma** | **GWAS lung function** |
| --- | --- | --- | --- | --- |
| hsa-miR-126-3p | ADAM9 | 8754 | - | - |
| hsa-miR-126-3p | ADM | 133 | - | - |
| hsa-miR-126-3p | BCL2 | 596 | - | - |
| hsa-miR-126-3p | CD97 | 976 | - | - |
| hsa-miR-126-3p | CRK | 1398 | - | - |
| hsa-miR-126-3p | CRKL | 1399 | - | - |
| hsa-miR-126-3p | CXCL12 | 6387 | - | - |
| hsa-miR-126-3p | CXCR4 | 7852 | 1 | - |
| hsa-miR-126-3p | DNMT1 | 1786 | - | - |
| hsa-miR-126-3p | EGFL7 | 51162 | - | - |
| hsa-miR-126-3p | FOXO3 | 2309 | - | - |
| hsa-miR-126-3p | HOXA9 | 3205 | - | - |
| hsa-miR-126-3p | IGFBP2 | 3485 | - | - |
| hsa-miR-126-3p | IRS1 | 3667 | - | - |
| hsa-miR-126-3p | KRAS | 3845 | - | - |
| hsa-miR-126-3p | LRP6 | 4040 | - | - |
| hsa-miR-126-3p | MERTK | 10461 | - | - |
| hsa-miR-126-3p | MMP7 | 4316 | - | - |
| hsa-miR-126-3p | NFKBIA | 4792 | - | - |
| hsa-miR-126-3p | PGR | 5241 | - | - |
| hsa-miR-126-3p | PIK3CG | 5294 | - | - |
| hsa-miR-126-3p | PIK3R2 | 5296 | - | - |
| hsa-miR-126-3p | PITPNC1 | 26207 | 1 | 1 |
| hsa-miR-126-3p | PLK2 | 10769 | - | - |
| hsa-miR-126-3p | PTPN7 | 5778 | - | - |
| hsa-miR-126-3p | RHOU | 58480 | - | - |
| hsa-miR-126-3p | SIRT1 | 23411 | - | - |
| hsa-miR-126-3p | SLC7A5 | 8140 | - | - |
| hsa-miR-126-3p | SOX2 | 6657 | - | - |
| hsa-miR-126-3p | SPRED1 | 161742 | - | - |
| hsa-miR-126-3p | TCF4 | 6925 | - | - |
| hsa-miR-126-3p | TEK | 7010 | - | - |
| hsa-miR-126-3p | TOM1 | 10043 | - | - |
| hsa-miR-126-3p | VCAM1 | 7412 | - | - |
| hsa-miR-126-3p | VEGFA | 7422 | - | - |
| hsa-miR-139-5p | CXCR4 | 7852 | 1 | - |
| hsa-miR-139-5p | FAM162A | 26355 | - | - |
| hsa-miR-139-5p | FOS | 2353 | - | - |
| hsa-miR-139-5p | HRAS | 3265 | - | - |
| hsa-miR-139-5p | IGF1R | 3480 | - | - |
| hsa-miR-139-5p | JUN | 3725 | - | - |
| hsa-miR-139-5p | MCL1 | 4170 | - | - |
| hsa-miR-139-5p | NFKB1 | 4790 | - | - |
| hsa-miR-139-5p | NOTCH1 | 4851 | - | - |
| hsa-miR-139-5p | NR5A2 | 2494 | - | - |
| hsa-miR-139-5p | PIK3CA | 5290 | - | - |
| hsa-miR-139-5p | RAP1B | 5908 | - | - |
| hsa-miR-139-5p | RHOT1 | 55288 | - | - |
| hsa-miR-139-5p | ROCK2 | 9475 | - | - |
| hsa-miR-139-5p | ZHX2 | 22882 | - | - |
| hsa-miR-142-3p | ABCG2 | 9429 | - | - |
| hsa-miR-142-3p | APC | 324 | - | - |
| hsa-miR-142-3p | ARNTL | 406 | - | - |
| hsa-miR-142-3p | CCNT2 | 905 | 1 | - |
| hsa-miR-142-3p | EGR2 | 1959 | - | - |
| hsa-miR-142-3p | HMGA1 | 3159 | - | - |
| hsa-miR-142-3p | HOXA10 | 3206 | - | - |
| hsa-miR-142-3p | HOXA7 | 3204 | - | - |
| hsa-miR-142-3p | HOXA9 | 3205 | - | - |
| hsa-miR-142-3p | HSPA1B | 3304 | - | - |
| hsa-miR-142-3p | IL1A | 3552 | - | - |
| hsa-miR-142-3p | LGR5 | 8549 | - | - |
| hsa-miR-142-3p | LPP | 4026 | - | - |
| hsa-miR-142-3p | LRRC32 | 2615 | 1 | - |
| hsa-miR-142-3p | PROM1 | 8842 | - | - |
| hsa-miR-142-3p | PTPN23 | 25930 | - | - |
| hsa-miR-142-3p | RAC1 | 5879 | - | - |
| hsa-miR-142-3p | ROCK2 | 9475 | - | - |
| hsa-miR-142-3p | TAB2 | 23118 | - | - |
| hsa-miR-142-3p | TGFBR1 | 7046 | - | - |
| hsa-miR-146b-5p | HNRNPD | 3184 | - | - |
| hsa-miR-146b-5p | IRAK1 | 3654 | - | - |
| hsa-miR-146b-5p | KIT | 3815 | - | - |
| hsa-miR-146b-5p | MMP16 | 4325 | - | - |
| hsa-miR-146b-5p | NFKB1 | 4790 | - | - |
| hsa-miR-146b-5p | PDGFRA | 5156 | - | - |
| hsa-miR-146b-5p | TLR4 | 7099 | - | - |
| hsa-miR-146b-5p | TRAF6 | 7189 | - | - |
| hsa-miR-146b-5p | UHRF1 | 29128 | - | - |
| hsa-miR-15b-5p | AXIN2 | 8313 | - | - |
| hsa-miR-15b-5p | BCL2 | 596 | - | - |
| hsa-miR-15b-5p | CCND1 | 595 | - | - |
| hsa-miR-15b-5p | CCNE1 | 898 | - | - |
| hsa-miR-15b-5p | EIF4A1 | 1973 | - | - |
| hsa-miR-15b-5p | FOXO1 | 2308 | - | - |
| hsa-miR-15b-5p | FUT2 | 2524 | - | - |
| hsa-miR-15b-5p | HNF1A | 6927 | - | - |
| hsa-miR-15b-5p | IFNG | 3458 | - | - |
| hsa-miR-15b-5p | KDR | 3791 | - | - |
| hsa-miR-15b-5p | PPM1D | 8493 | - | - |
| hsa-miR-15b-5p | PURA | 5813 | - | - |
| hsa-miR-15b-5p | RECK | 8434 | - | - |
| hsa-miR-15b-5p | SMURF1 | 57154 | - | - |
| hsa-miR-15b-5p | VEGFA | 7422 | - | - |
| hsa-miR-16-5p | ACVR2A | 92 | - | - |
| hsa-miR-16-5p | AKT3 | 10000 | - | - |
| hsa-miR-16-5p | ARL2 | 402 | - | - |
| hsa-miR-16-5p | AXIN2 | 8313 | - | - |
| hsa-miR-16-5p | BCL2 | 596 | - | - |
| hsa-miR-16-5p | BDNF | 627 | - | - |
| hsa-miR-16-5p | BIRC5 | 332 | - | - |
| hsa-miR-16-5p | BMI1 | 648 | - | - |
| hsa-miR-16-5p | BRCA1 | 672 | - | - |
| hsa-miR-16-5p | CADM1 | 23705 | - | - |
| hsa-miR-16-5p | CAPRIN1 | 4076 | - | - |
| hsa-miR-16-5p | CCND1 | 595 | - | - |
| hsa-miR-16-5p | CCND3 | 896 | - | - |
| hsa-miR-16-5p | CCNE1 | 898 | - | - |
| hsa-miR-16-5p | CDK6 | 1021 | - | - |
| hsa-miR-16-5p | CDS2 | 8760 | - | - |
| hsa-miR-16-5p | CHEK1 | 1111 | - | - |
| hsa-miR-16-5p | CHUK | 1147 | - | - |
| hsa-miR-16-5p | FGFR1 | 2260 | - | - |
| hsa-miR-16-5p | HDGF | 3068 | - | - |
| hsa-miR-16-5p | HMGA1 | 3159 | - | - |
| hsa-miR-16-5p | HMGA2 | 8091 | - | - |
| hsa-miR-16-5p | IFNG | 3458 | - | - |
| hsa-miR-16-5p | IGF1R | 3480 | - | - |
| hsa-miR-16-5p | KDR | 3791 | - | - |
| hsa-miR-16-5p | KRAS | 3845 | - | - |
| hsa-miR-16-5p | MAP7 | 9053 | - | - |
| hsa-miR-16-5p | MTOR | 2475 | - | - |
| hsa-miR-16-5p | MYB | 4602 | - | - |
| hsa-miR-16-5p | NCOR2 | 9612 | - | - |
| hsa-miR-16-5p | PIM1 | 5292 | - | - |
| hsa-miR-16-5p | PPM1D | 8493 | - | - |
| hsa-miR-16-5p | PRDM4 | 11108 | - | - |
| hsa-miR-16-5p | PTGS2 | 5743 | - | - |
| hsa-miR-16-5p | PURA | 5813 | - | - |
| hsa-miR-16-5p | RAF1 | 5894 | - | - |
| hsa-miR-16-5p | RECK | 8434 | - | - |
| hsa-miR-16-5p | RICTOR | 253260 | - | - |
| hsa-miR-16-5p | RPS6KB1 | 6198 | - | - |
| hsa-miR-16-5p | SLC6A4 | 6532 | - | - |
| hsa-miR-16-5p | SOX5 | 6660 | - | - |
| hsa-miR-16-5p | SOX6 | 55553 | - | - |
| hsa-miR-16-5p | TP53 | 7157 | - | - |
| hsa-miR-16-5p | TPPP3 | 51673 | - | - |
| hsa-miR-16-5p | UNG | 7374 | - | - |
| hsa-miR-16-5p | VEGFA | 7422 | - | - |
| hsa-miR-16-5p | WEE1 | 7465 | - | - |
| hsa-miR-16-5p | WNT3A | 89780 | - | - |
| hsa-miR-16-5p | WNT4 | 54361 | - | - |
| hsa-miR-16-5p | YAP1 | 10413 | - | - |
| hsa-miR-16-5p | ZYX | 7791 | - | - |
| hsa-miR-186-5p | AKAP12 | 9590 | - | - |
| hsa-miR-186-5p | CSNK2A1 | 1457 | - | - |
| hsa-miR-186-5p | FOXO1 | 2308 | - | - |
| hsa-miR-186-5p | NCSTN | 23385 | - | - |
| hsa-miR-186-5p | P2RX7 | 5027 | - | - |
| hsa-miR-186-5p | PTTG1 | 9232 | - | - |
| hsa-miR-191-5p | CCND2 | 894 | - | - |
| hsa-miR-191-5p | CDK6 | 1021 | - | - |
| hsa-miR-191-5p | CEBPB | 1051 | 1 | - |
| hsa-miR-191-5p | CTDSP2 | 10106 | - | - |
| hsa-miR-191-5p | EGR1 | 1958 | - | - |
| hsa-miR-191-5p | IL1A | 3552 | - | - |
| hsa-miR-191-5p | LRRC8A | 56262 | - | - |
| hsa-miR-191-5p | MDM4 | 4194 | - | - |
| hsa-miR-191-5p | NDST1 | 3340 | - | - |
| hsa-miR-191-5p | SATB1 | 6304 | - | - |
| hsa-miR-191-5p | SLC16A2 | 6567 | - | - |
| hsa-miR-191-5p | SOX4 | 6659 | - | 1 |
| hsa-miR-191-5p | TMC7 | 79905 | - | - |
| hsa-miR-191-5p | YBX3 | 8531 | - | - |
| hsa-miR-203a-3p | ABCE1 | 6059 | - | - |
| hsa-miR-203a-3p | ABL1 | 25 | - | - |
| hsa-miR-203a-3p | AKT2 | 208 | - | - |
| hsa-miR-203a-3p | ASAP1 | 50807 | 1 | - |
| hsa-miR-203a-3p | ATM | 472 | - | - |
| hsa-miR-203a-3p | BCL2L2 | 599 | - | - |
| hsa-miR-203a-3p | BIRC5 | 332 | - | - |
| hsa-miR-203a-3p | BMI1 | 648 | - | - |
| hsa-miR-203a-3p | CASK | 8573 | - | - |
| hsa-miR-203a-3p | CAV1 | 857 | - | - |
| hsa-miR-203a-3p | CBLL1 | 79872 | - | - |
| hsa-miR-203a-3p | CDK6 | 1021 | - | - |
| hsa-miR-203a-3p | CREB1 | 1385 | - | - |
| hsa-miR-203a-3p | DLX5 | 1749 | - | - |
| hsa-miR-203a-3p | E2F1 | 1869 | - | - |
| hsa-miR-203a-3p | E2F3 | 1871 | - | - |
| hsa-miR-203a-3p | EDNRA | 1909 | - | - |
| hsa-miR-203a-3p | EYA4 | 2070 | - | - |
| hsa-miR-203a-3p | GDAP1 | 54332 | - | - |
| hsa-miR-203a-3p | IFIT1 | 3434 | - | - |
| hsa-miR-203a-3p | IL24 | 11009 | - | - |
| hsa-miR-203a-3p | JUN | 3725 | - | - |
| hsa-miR-203a-3p | KIF2A | 3796 | - | - |
| hsa-miR-203a-3p | KIF5B | 3799 | - | - |
| hsa-miR-203a-3p | LASP1 | 3927 | - | - |
| hsa-miR-203a-3p | LIFR | 3977 | - | - |
| hsa-miR-203a-3p | MMP10 | 4319 | - | - |
| hsa-miR-203a-3p | MYD88 | 4615 | - | - |
| hsa-miR-203a-3p | PIK3CA | 5290 | - | - |
| hsa-miR-203a-3p | PLD2 | 5338 | - | - |
| hsa-miR-203a-3p | RAN | 5901 | - | - |
| hsa-miR-203a-3p | RAPH1 | 65059 | - | - |
| hsa-miR-203a-3p | RUNX2 | 860 | - | - |
| hsa-miR-203a-3p | SMAD4 | 4089 | - | - |
| hsa-miR-203a-3p | SNAI1 | 6615 | - | - |
| hsa-miR-203a-3p | SNAI2 | 6591 | - | - |
| hsa-miR-203a-3p | SOCS3 | 9021 | - | - |
| hsa-miR-203a-3p | SOCS6 | 9306 | - | - |
| hsa-miR-203a-3p | SRC | 6714 | - | - |
| hsa-miR-203a-3p | TJP2 | 9414 | - | - |
| hsa-miR-203a-3p | TNF | 7124 | - | - |
| hsa-miR-203a-3p | TP63 | 8626 | - | - |
| hsa-miR-203a-3p | UVRAG | 7405 | - | - |
| hsa-miR-203a-3p | VEGFA | 7422 | - | - |
| hsa-miR-203a-3p | ZEB2 | 9839 | - | - |
| hsa-miR-203a-3p | ZNF148 | 7707 | - | - |
| hsa-miR-206 | ACTL6A | 86 | - | - |
| hsa-miR-206 | CCND1 | 595 | - | - |
| hsa-miR-206 | CCND2 | 894 | - | - |
| hsa-miR-206 | CDK4 | 1019 | - | - |
| hsa-miR-206 | ESR1 | 2099 | - | - |
| hsa-miR-206 | FSTL1 | 11167 | - | - |
| hsa-miR-206 | G6PD | 2539 | - | - |
| hsa-miR-206 | GJA1 | 2697 | - | - |
| hsa-miR-206 | GPD2 | 2820 | 1 | - |
| hsa-miR-206 | KRAS | 3845 | - | - |
| hsa-miR-206 | MET | 4233 | - | - |
| hsa-miR-206 | NOTCH3 | 4854 | - | - |
| hsa-miR-206 | NR1H3 | 10062 | - | - |
| hsa-miR-206 | PAX3 | 5077 | 1 | - |
| hsa-miR-206 | PGD | 5226 | - | - |
| hsa-miR-206 | TAC1 | 6863 | - | - |
| hsa-miR-206 | TKT | 7086 | - | - |
| hsa-miR-206 | UTRN | 7402 | - | - |
| hsa-miR-206 | VAMP2 | 6844 | - | - |
| hsa-miR-26a-5p | ABCA1 | 19 | - | - |
| hsa-miR-26a-5p | ACVR1 | 90 | - | - |
| hsa-miR-26a-5p | ADAM17 | 6868 | - | - |
| hsa-miR-26a-5p | AMACR | 23600 | - | - |
| hsa-miR-26a-5p | ARL4C | 10123 | - | - |
| hsa-miR-26a-5p | ATM | 472 | - | - |
| hsa-miR-26a-5p | BAG4 | 9530 | - | - |
| hsa-miR-26a-5p | CCND2 | 894 | - | - |
| hsa-miR-26a-5p | CCNE1 | 898 | - | - |
| hsa-miR-26a-5p | CCNE2 | 9134 | - | - |
| hsa-miR-26a-5p | CDK6 | 1021 | - | - |
| hsa-miR-26a-5p | CHD1 | 1105 | - | - |
| hsa-miR-26a-5p | CHEK1 | 1111 | - | - |
| hsa-miR-26a-5p | CKS2 | 1164 | - | - |
| hsa-miR-26a-5p | CPEB2 | 132864 | - | - |
| hsa-miR-26a-5p | CPEB3 | 22849 | - | - |
| hsa-miR-26a-5p | CPEB4 | 80315 | - | - |
| hsa-miR-26a-5p | DNMT3B | 1789 | - | - |
| hsa-miR-26a-5p | ESR1 | 2099 | - | - |
| hsa-miR-26a-5p | EZH2 | 2146 | - | - |
| hsa-miR-26a-5p | FGF9 | 2254 | - | - |
| hsa-miR-26a-5p | GDAP1 | 54332 | - | - |
| hsa-miR-26a-5p | GSK3B | 2932 | - | - |
| hsa-miR-26a-5p | HGF | 3082 | - | - |
| hsa-miR-26a-5p | HMGA1 | 3159 | - | - |
| hsa-miR-26a-5p | HMGA2 | 8091 | - | - |
| hsa-miR-26a-5p | IFNB1 | 3456 | - | - |
| hsa-miR-26a-5p | IL6 | 3569 | - | - |
| hsa-miR-26a-5p | LIN28B | 389421 | - | - |
| hsa-miR-26a-5p | MAP3K2 | 10746 | - | - |
| hsa-miR-26a-5p | MCL1 | 4170 | - | - |
| hsa-miR-26a-5p | MTDH | 92140 | - | - |
| hsa-miR-26a-5p | NOS2 | 4843 | - | - |
| hsa-miR-26a-5p | PHB | 5245 | - | - |
| hsa-miR-26a-5p | PIK3C2A | 5286 | - | - |
| hsa-miR-26a-5p | PLAG1 | 5324 | - | - |
| hsa-miR-26a-5p | PRKCD | 5580 | - | - |
| hsa-miR-26a-5p | PTEN | 5728 | - | - |
| hsa-miR-26a-5p | RB1 | 5925 | - | - |
| hsa-miR-26a-5p | RCBTB1 | 55213 | - | - |
| hsa-miR-26a-5p | SERBP1 | 26135 | - | - |
| hsa-miR-26a-5p | SMAD1 | 4086 | - | - |
| hsa-miR-26a-5p | SMAD4 | 4089 | - | - |
| hsa-miR-26a-5p | TDG | 6996 | - | - |
| hsa-miR-26a-5p | TET2 | 54790 | - | 1 |
| hsa-miR-26a-5p | WEE1 | 7465 | - | - |
| hsa-miR-26a-5p | ZCCHC11 | 23318 | - | - |
| hsa-miR-301a-3p | BCL2L11 | 10018 | - | - |
| hsa-miR-301a-3p | MEOX2 | 4223 | - | - |
| hsa-miR-301a-3p | NKRF | 55922 | - | - |
| hsa-miR-301a-3p | PTEN | 5728 | - | - |
| hsa-miR-301a-3p | RUNX3 | 864 | - | - |
| hsa-miR-301a-3p | SERPINE1 | 5054 | - | - |
| hsa-miR-301a-3p | SMAD4 | 4089 | - | - |
| hsa-miR-30b-5p | ATG12 | 9140 | - | - |
| hsa-miR-30b-5p | BCL2 | 596 | - | - |
| hsa-miR-30b-5p | BCL6 | 604 | - | 1 |
| hsa-miR-30b-5p | BCL9 | 607 | - | - |
| hsa-miR-30b-5p | BECN1 | 8678 | - | - |
| hsa-miR-30b-5p | CAT | 847 | - | - |
| hsa-miR-30b-5p | CCNE2 | 9134 | - | - |
| hsa-miR-30b-5p | DLL4 | 54567 | - | - |
| hsa-miR-30b-5p | DNMT1 | 1786 | - | - |
| hsa-miR-30b-5p | ERG | 2078 | - | - |
| hsa-miR-30b-5p | NOTCH1 | 4851 | - | - |
| hsa-miR-30b-5p | PDGFRB | 5159 | - | - |
| hsa-miR-30b-5p | RUNX2 | 860 | - | - |
| hsa-miR-30b-5p | SERPINE1 | 5054 | - | - |
| hsa-miR-30b-5p | SIX1 | 6495 | - | - |
| hsa-miR-30b-5p | SMAD1 | 4086 | - | - |
| hsa-miR-30b-5p | SNAI1 | 6615 | - | - |
| hsa-miR-30b-5p | SOCS1 | 8651 | - | - |
| hsa-miR-30b-5p | TP53 | 7157 | - | - |
| hsa-miR-331-3p | DOHH | 83475 | - | - |
| hsa-miR-331-3p | E2F1 | 1869 | - | - |
| hsa-miR-331-3p | ERBB2 | 2064 | - | - |
| hsa-miR-331-3p | FHIT | 2272 | - | - |
| hsa-miR-331-3p | HOTAIR | 100124700 | - | - |
| hsa-miR-331-3p | NRP2 | 8828 | 1 | - |
| hsa-miR-331-3p | PHLPP1 | 23239 | - | - |
| hsa-miR-342-3p | BMP7 | 655 | - | - |
| hsa-miR-342-3p | DNMT1 | 1786 | - | - |
| hsa-miR-342-3p | GEMIN4 | 50628 | - | - |
| hsa-miR-342-3p | ID4 | 3400 | - | - |
| hsa-miR-342-3p | SREBF1 | 6720 | - | - |
| hsa-miR-342-3p | SREBF2 | 6721 | - | - |
| hsa-miR-374a-5p | ATM | 472 | - | - |
| hsa-miR-374a-5p | CEBPB | 1051 | 1 | - |
| hsa-miR-374a-5p | DICER1 | 23405 | - | - |
| hsa-miR-374a-5p | GADD45A | 1647 | - | - |
| hsa-miR-374a-5p | SRCIN1 | 80725 | - | - |
| hsa-miR-374a-5p | WIF1 | 11197 | - | - |
| hsa-miR-374a-5p | WNT5A | 7474 | - | - |
| hsa-miR-409-3p | ANG | 283 | - | - |
| hsa-miR-409-3p | FGB | 2244 | - | - |
| hsa-miR-409-3p | IFNG | 3458 | - | - |
| hsa-miR-409-3p | MET | 4233 | - | - |
| hsa-miR-409-3p | PHF10 | 55274 | - | - |
| hsa-miR-409-3p | RDX | 5962 | - | - |
| hsa-miR-454-3p | SMAD4 | 4089 | - | - |
| hsa-miR-484 | FIS1 | 51024 | - | - |
| hsa-miR-942-5p | IFI27 | 3429 | - | - |
| hsa-miR-106b-5p | APC | 324 | - | - |
| hsa-miR-106b-5p | APP | 351 | - | - |
| hsa-miR-106b-5p | ATG16L1 | 55054 | - | - |
| hsa-miR-106b-5p | BCL2L11 | 10018 | - | - |
| hsa-miR-106b-5p | CASP7 | 840 | - | - |
| hsa-miR-106b-5p | CASP8 | 841 | - | - |
| hsa-miR-106b-5p | CCND1 | 595 | - | - |
| hsa-miR-106b-5p | CCND2 | 894 | - | - |
| hsa-miR-106b-5p | CDKN1A | 1026 | - | - |
| hsa-miR-106b-5p | E2F1 | 1869 | - | - |
| hsa-miR-106b-5p | E2F5 | 1875 | - | - |
| hsa-miR-106b-5p | EOMES | 8320 | - | - |
| hsa-miR-106b-5p | ITCH | 83737 | - | - |
| hsa-miR-106b-5p | JAK1 | 3716 | - | - |
| hsa-miR-106b-5p | KAT2B | 8850 | - | - |
| hsa-miR-106b-5p | MFN2 | 9927 | - | - |
| hsa-miR-106b-5p | MMP2 | 4313 | - | - |
| hsa-miR-106b-5p | PKD2 | 5311 | - | - |
| hsa-miR-106b-5p | PTEN | 5728 | - | - |
| hsa-miR-106b-5p | PURA | 5813 | - | - |
| hsa-miR-106b-5p | RB1 | 5925 | - | - |
| hsa-miR-106b-5p | RBL1 | 5933 | - | - |
| hsa-miR-106b-5p | RBL2 | 5934 | - | - |
| hsa-miR-106b-5p | RUNX3 | 864 | - | - |
| hsa-miR-106b-5p | SMAD7 | 4092 | - | - |
| hsa-miR-106b-5p | STAT3 | 6774 | - | - |
| hsa-miR-106b-5p | TCEAL1 | 9338 | - | - |
| hsa-miR-106b-5p | TWIST1 | 7291 | 1 | - |
| hsa-miR-106b-5p | VEGFA | 7422 | - | - |
| hsa-miR-106b-5p | WEE1 | 7465 | - | - |
| hsa-miR-106b-5p | ZBTB4 | 57659 | - | - |
| hsa-miR-27b-3p | ABCA1 | 19 | - | - |
| hsa-miR-27b-3p | ADORA2B | 136 | - | - |
| hsa-miR-27b-3p | CCNA2 | 890 | - | - |
| hsa-miR-27b-3p | CCNT1 | 904 | - | - |
| hsa-miR-27b-3p | CREB1 | 1385 | - | - |
| hsa-miR-27b-3p | CYP1B1 | 1545 | - | - |
| hsa-miR-27b-3p | CYP3A4 | 1576 | - | - |
| hsa-miR-27b-3p | DPYD | 1806 | - | - |
| hsa-miR-27b-3p | EDNRA | 1909 | - | - |
| hsa-miR-27b-3p | EYA4 | 2070 | - | - |
| hsa-miR-27b-3p | KHSRP | 8570 | - | - |
| hsa-miR-27b-3p | MFF | 56947 | - | - |
| hsa-miR-27b-3p | MMP13 | 4322 | 1 | - |
| hsa-miR-27b-3p | NOTCH1 | 4851 | - | - |
| hsa-miR-27b-3p | PAX3 | 5077 | 1 | - |
| hsa-miR-27b-3p | PAX7 | 5081 | - | - |
| hsa-miR-27b-3p | PHB | 5245 | - | - |
| hsa-miR-27b-3p | PPARG | 5468 | - | - |
| hsa-miR-27b-3p | PSAP | 5660 | 1 | - |
| hsa-miR-27b-3p | RET | 5979 | - | - |
| hsa-miR-27b-3p | SEMA6A | 57556 | - | - |
| hsa-miR-27b-3p | SHC1 | 6464 | - | - |
| hsa-miR-27b-3p | ST14 | 6768 | - | - |
| hsa-miR-27b-3p | THBS1 | 7057 | - | - |
| hsa-miR-27b-3p | THBS2 | 7058 | - | - |
| hsa-miR-27b-3p | VDR | 7421 | - | - |
| hsa-miR-27b-3p | VEGFC | 7424 | - | - |
| hsa-miR-27b-3p | WEE1 | 7465 | - | - |
| hsa-miR-320a | AQP1 | 358 | - | - |
| hsa-miR-320a | AQP4 | 361 | - | - |
| hsa-miR-320a | ARF1 | 375 | - | - |
| hsa-miR-320a | BANP | 54971 | - | - |
| hsa-miR-320a | BMI1 | 648 | - | - |
| hsa-miR-320a | GNAI1 | 2770 | - | - |
| hsa-miR-320a | HSPB6 | 126393 | - | - |
| hsa-miR-320a | IGF1R | 3480 | - | - |
| hsa-miR-320a | ITGB3 | 3690 | - | - |
| hsa-miR-320a | MAPK1 | 5594 | - | - |
| hsa-miR-320a | MCL1 | 4170 | - | - |
| hsa-miR-320a | NFATC3 | 4775 | - | - |
| hsa-miR-320a | NPR1 | 4881 | - | - |
| hsa-miR-320a | NRP1 | 8829 | - | - |
| hsa-miR-320a | POLR3D | 661 | - | - |
| hsa-miR-320a | PTEN | 5728 | - | - |
| hsa-miR-320a | RAC1 | 5879 | - | - |
| hsa-miR-320a | TFRC | 7037 | - | - |
| hsa-miR-320a | TRPC5 | 7224 | - | - |
| hsa-miR-340-5p | HNRNPA2B1 | 3181 | - | - |
| hsa-miR-340-5p | KRAS | 3845 | - | - |
| hsa-miR-340-5p | MECP2 | 4204 | - | - |
| hsa-miR-340-5p | MET | 4233 | - | - |
| hsa-miR-340-5p | PTBP1 | 5725 | - | - |
| hsa-miR-340-5p | RHOA | 387 | - | - |
| hsa-miR-340-5p | ROCK1 | 6093 | - | - |
| hsa-miR-340-5p | SOX2 | 6657 | - | - |
| hsa-miR-376c-3p | ACVR1C | 130399 | - | - |
| hsa-miR-376c-3p | GRB2 | 2885 | - | - |
| hsa-miR-376c-3p | IGF1R | 3480 | - | - |
| hsa-miR-376c-3p | TGFA | 7039 | - | - |
| hsa-miR-376c-3p | TGFBR1 | 7046 | - | - |
| hsa-miR-645 | IFIT2 | 3433 | - | - |
| hsa-miR-1290 | no target |  |  |  |
| hsa-miR-660-5p | no target |  |  |  |
| hsa-miR-223-5p | no target |  |  |  |
| hsa-miR-339-3p | no target |  |  |  |
|  |  |  |  |  |
